# Annexin A7 Regulates Endometrial Receptivity

**DOI:** 10.3389/fcell.2020.00770

**Published:** 2020-08-14

**Authors:** Md Alauddin, Madhuri S. Salker, Anja T. Umbach, Janet Rajaxavier, Toshiyuki Okumura, Yogesh Singh, Anna Wagner, Sara Y. Brucker, Diethelm Wallwiener, Jan J. Brosens, Florian Lang

**Affiliations:** ^1^Department of Women’s Health, Eberhard Karls University of Tübingen, Tübingen, Germany; ^2^Department of Physiology, Eberhard Karls University of Tübingen, Tübingen, Germany; ^3^Department of Obstetrics and Gynecology, Juntendo University School of Medicine, Tokyo, Japan; ^4^Institute of Medical Genetics and Applied Genomics, Eberhard Karls University of Tübingen, Tübingen, Germany; ^5^Division of Biomedical Sciences, Warwick Medical School, Coventry, United Kingdom; ^6^Tommy’s National Centre for Miscarriage Research, University Hospitals Coventry and Warwickshire NHS Trust, Coventry, United Kingdom

**Keywords:** endometrium, COX2, implantation, pregnancy, PGE_2_

## Abstract

A limited window of receptivity is a prerequisite of reproductive success. Indispensable receptivity genes include cyclooxygenase 2 (COX2), an enzyme accomplishing formation of prostaglandin E_2_ (PGE_2_). A powerful regulator of PGE_2_ formation is Annexin A7 (*ANXA7*). The present study thus explored whether ANXA7 impacts on implantation and fertility. Here we show that *ANXA7* is expressed in endometrial tissue and increases upon decidual transformation of human endometrial stromal cells (HESCs) in a time-dependent manner. Silencing ANXA7 significantly decreased the expression of *PRL* and *IGFBP1*, canonical decidual marker genes, but enhances COX2 and PGE_2_ levels. Genetic knockout of *AnxA7* in mice significantly increases the number of implantation sites and litter sizes. Further, analysis of human endometrial biopsies showed that *ANXA7* transcript and protein levels are decreased during the midluteal window of implantation in women suffering from recurrent pregnancy loss (RPL) when compared to subfertile patients. Taken together, the data indicate that ANXA7 has a conserved role in regulating endometrial receptivity and implantation.

## Introduction

Following the post-ovulatory rise in progesterone levels, human endometrial stromal cells (HESCs) undergo extensive biochemical and morphological reprogramming, a process known as decidualization, in preparation for pregnancy (Koot et al., 2012; Gellersen and Brosens, 2014). While this process is initiated during the mid-luteal phase of the cycle, the emergence of morphologically decidualized cells at the start of the late-luteal phase marks the end of the implantation window, defined as the limited period during which a developmentally competent blastocyst can implant (Cha et al., 2012; Koot et al., 2012; Gellersen and Brosens, 2014; Macklon and Brosens, 2014). Decidualization is thus essential for the establishment of uterine receptivity, post-implantation embryo survival, and placentation. Key factors involved in endometrial receptivity and embryo implantation include prostaglandin (PG) production (namely PGE_2_) by cyclooxygenase 2 (COX2; PTGS2) (Cha et al., 2012; Ruan et al., 2012).

Prostaglandin synthesis starts with the formation of arachidonic acid (AA) from membrane phospholipids mediated by phospholipase A_2_ (PLA_2_) (Vilella et al., 2013). Subsequently, COX2, *via* its peroxidase activity, converts AA into PGs, including PGE_2_ (Leslie, 2004; Cha et al., 2012; Ruan et al., 2012; Vilella et al., 2013). In the endometrium, COX2 levels and PGE_2_ production increase during the luteal phase of the menstrual cycle and during early pregnancy (Ye et al., 2018). Compromised endometrial prostaglandin synthesis is observed in women with repeated implantation failure (Achache et al., 2010). Importantly, loss of COX2 or PLA_2_ can result in infertility due to abnormalities of ovulation, implantation, and decidualization in mice (Lim et al., 1997; Vilella et al., 2013; Salker et al., 2018).

As shown in other cell types, COX2, PLA_2_ and thus PGE_2_ formation are strictly controlled by multiple factors, including Annexin A7 (synexin or ANXA7) (Scott et al., 1999; Harper and Tyson-Capper, 2008). Annexins are a family of evolutionary calcium-dependent phospholipids binding proteins, which are found in almost all tissues and cell types (Benz and Hofmann, 1997; Gerke and Moss, 2002; Guo et al., 2013). In humans, twelve annexin subfamilies have been described (Annexin A1-11 and Annexin 13) (Benz and Hofmann, 1997; Gerke and Moss, 2002; Guo et al., 2013). These proteins are mainly distributed in the inner surface of the plasma membrane and have important roles in regulation of cytoskeletal activity, cell adhesion, membrane receptor regulation, membrane transport and mitosis (Liu et al., 2020). ANXA7 was the first annexin protein to be described (Benz and Hofmann, 1997; Gerke and Moss, 2002). It was first isolated as a factor that mediates aggregation of chromaffin granules and fusion of phospholipids membranes in the presence of Ca^2+^ and in Ca^2+^-GTP-dependent secretion (Herr et al., 2001; Guo et al., 2013). ANXA7 differs from other members as it possesses and extra-long amino terminus (Camors et al., 2005; Rick et al., 2005) and alternative splicing generates two isoforms with molecular weights of 47 kDa and 51 kDa. Most tissues harbor the 47 kDa isoform, while the larger isoform is expressed in skeletal muscle, heart, and brain (Camors et al., 2005; Rick et al., 2005). ANXA7 has a unique architecture that allows it to dock in a Ca^2+^ dependent manner onto the phospholipid cell membrane; this prevents PLA_2_ from binding to the membrane, thus inhibiting PGE_2_ formation (Herr et al., 2001, 2003; Clemen et al., 2003; Rick et al., 2005; Schrickel et al., 2007; Lang et al., 2009, 2010; Guo et al., 2013). Consequently, ANXA7 influences many physiological processes including secretion, hormone release, cell survival, cell volume, cardiac remodeling, gastric acid secretion, and inflammation by inhibiting PLA_2_ and PGE_2_ formation (Lang et al., 2009; Luo et al., 2015). Overexpression of ANXA7 is associated with aggressive tumors by accelerating cell cycle progression and proliferation (Liu et al., 2020).

Knockout of *AnxA7* in mice was initially reported to be lethal on embryonic day 10 (Srivastava et al., 1999). A second attempt by Herr et al. (2001) yielded viable *AnxA7* knockout mice (Herr et al., 2001). In the mouse line described in the latter study, the neo cassette was inserted directly into exon 8 of the *AnxA7* gene (Herr et al., 2001). Srivastava et al. (1999) replaced part of intron 5 and exon 6, an exon transcribed selectively in striated muscle and brain. Hence, the results of the two knockout models might be due to alterations in the expression of other genes in the vicinity of the integration site.

We speculated that ANXA7 may modulate endometrial receptivity in view of its role in regulating prostaglandin synthesis. However, to the best of our knowledge, little is known about the impact of ANXA7 on early implantation events. The present study thus explored the impact of ANXA7 on embryo implantation and fertility. We examined the expression of ANXA7 upon decidual transformation of HESCs. We demonstrated that ANXA7 knockdown impairs the induction of cardinal decidual markers prolactin (PRL) and insulin-like growth factor binding protein 1 (IGFBP1), but enhances COX2 and PGE_2_ levels in human endometrium. We report that litter sizes are significantly larger in *AnxA7^–/–^* knockout females when compared to wild-type littermates. Finally, we disclose that ANXA7 levels are higher in women subfertility (SF) when compared to women with recurrent pregnancy loss (RPL).

## Results

### Expression of ANXA7 in Human Endometrium

**FIGURE 1:**
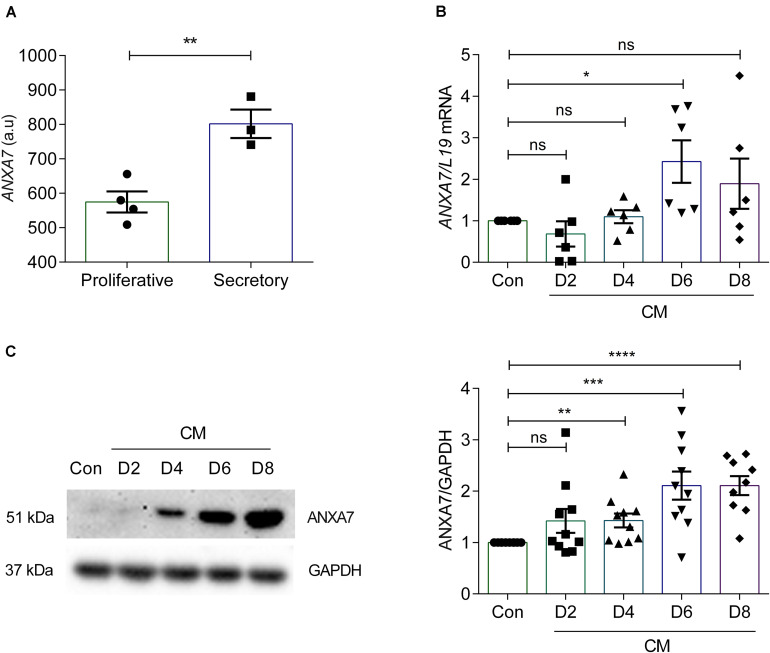
ANXA7 level in human endometrium and human endometrial stromal cells (HESCs). **(A)** *ANXA7* gene expression is upregulated in secretory phase in human endometrium (Gene Expression Omnibus; GEO ID 24464767). **(B)** Arithmetic means ± SEM (*n* = 6) of *ANXA7* mRNA levels in HESCs, decidualized with 0.5 μM 8-Br-cAMP and 1 μM MPA (CM) up to 8 days. **(C)** Original Western blot (left) and arithmetic means ± SEM (*n* = 10; right) of *ANXA7* in HESCs decidualized with 0.5 μM 8-Br-cAMP and 1 μM MPA (CM) up to 8 days. Student’s *t*-test was used to calculate statistical significance. *P < 0.05, **P < 0.01, ***P < 0.001, ****P < 0.0001.

According to the Human Protein Atlas, ANXA7 is expressed in the endometrial glands and stroma and luminal epithelium (Uhlen et al., 2005). To investigate *ANXA7* mRNA levels in cycling endometrium we performed *in silico* analysis of gene expression data obtained in human endometrium across the cycle (GEO Profiles ID: 24464767). Endometrial *ANXA7* transcript levels increased from the proliferative to the early secretory phase of the cycle (Figure 1A). To investigate whether ANXA7 plays a role in decidualization, primary HESCs were decidualized with 0.5 μM 8-br-cAMP and 1 μM MPA (CM) for a total of 8 days. ANXA7 expression was examined at both mRNA and protein levels. Analysis of independent primary cultures demonstrated that ANXA7 (at both mRNA and protein levels) remained low during the initial pro-inflammatory decidual phase (days 2–4; aligned to the implantation window) (Salker et al., 2012a; Lucas et al., 2020) before rising around days 6–8, which coincides with the emergence of specialized decidual cells (aligned to the refractory period *in vivo*) (Salker et al., 2012a; Lucas et al., 2020; Figures 1B,C and Supplementary Figure S1).

### Impact of ANXA7 Knockdown on Decidual Marker Genes

**FIGURE 2:**
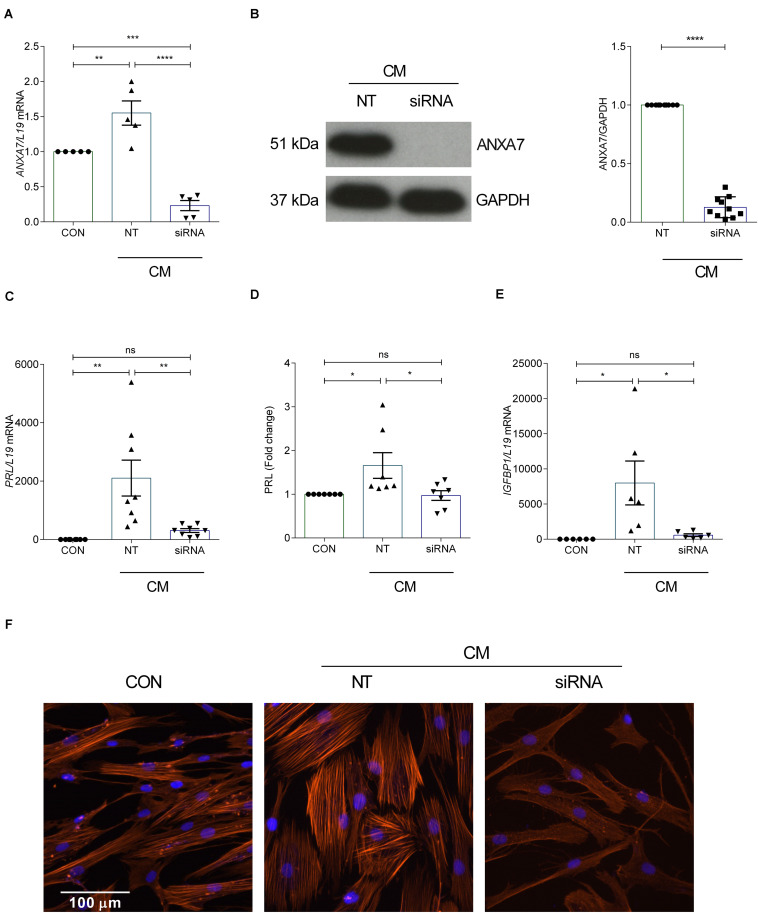
Loss of ANXA7 reduces key decidual markers. HESCs were transfected by non-targeting (NT) or siRNA *ANXA7* and then decidualized for 6 days with 0.5 μM 8-Br-cAMP and 1 μM MPA (CM). **(A)** Arithmetic means ± SEM (*n* = 5) of *ANXA7* mRNA levels. **(B)** Original Western blot (left) and arithmetic means ± SEM (*n* = 10; right) of ANXA7 protein abundance. Arithmetic means ± SEM (*n* = 6–8) of **(C)** *PRL transcript* and **(D)** secreted PRL levels and **(E)** *IGFBP* expression levels in decidualizing HESCs. **(F)** Immunofluorescence images of F-actin (Phalloidin, red) in undifferentiated HESCs, decidualised or with siRNA targeting ANXA7. Nuclei are stained blue with DAPI (*n* = 3). Scale bar (100 μM). ANOVA or Student’s *t*-test was used to calculate statistical significance when appropriate. *P < 0.05, **P < 0.01, ***P < 0.001, ****P < 0.0001.

Decidualization denotes the differentiation process by which HESCs acquire a specialized secretory phenotype. *PRL* and *IGFBP1* are canonical marker genes widely used to assess the quality of the decidual response in HESCs (Saleh et al., 2011). In order to test whether ANXA7 influences the expression of decidual marker genes, primary cultures were first transfected with NT or siRNA targeting ANXA7 and then decidualized with CM for 6 days. As seen in Figures 2A,B and Supplementary Figure S2, siRNA-mediated knockdown decreased ANXA7 expression at both transcript and protein level by 85.05% and 87.23%, respectively. ANXA7 knockdown also inhibited *PRL* and *IGFBP1* mRNA levels in decidualizing cultures as well as secreted PRL levels (Figures 2C–E). The differentiation-associated changes in the actin cytoskeleton by phalloidin staining of filamentous actin (F-actin) was also reduced in cells targeted with siRNA-ANXA7 (Figure 2F).

### Downregulating ANXA7 Increases COX2 Levels

**FIGURE 3:**
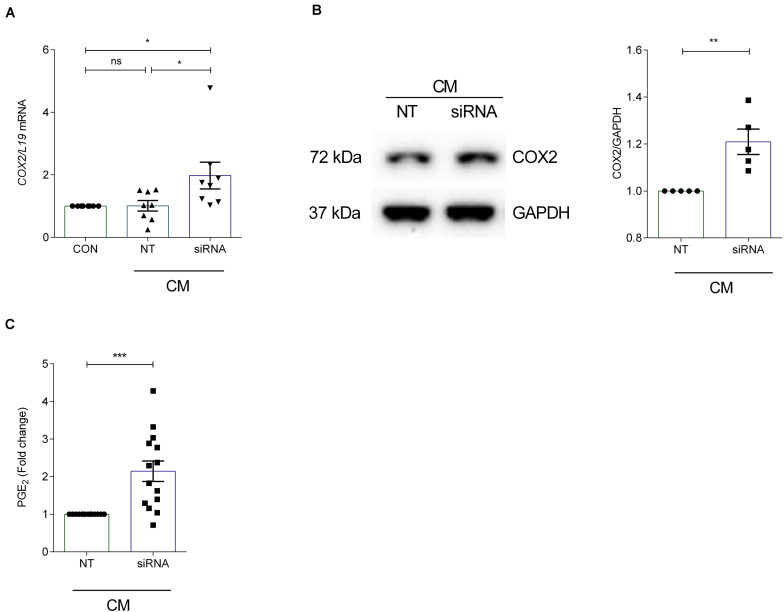
Silencing of *ANXA7* upregulates COX2 and PGE_2_ in HESCs. HESCs were transfected by non-targeting (NT) or siRNA targeting *ANXA7* and then decidualized for 6 days with 0.5 μM 8-Br-cAMP and 1 μM MPA (CM). **(A)** Arithmetic means ± SEM (*n* = 8) of *COX2* mRNA levels. **(B)** Original Western blot (left) and arithmetic means ± SEM (*n* = 5, right) of COX2 protein abundance in HESCs. **(C)** Arithmetic means ± SEM (*n* = 14) of PGE_2_ levels measured by ELISA. ANOVA or Student’s *t*-test was used to calculate statistical significance when appropriate. *P < 0.05, **P < 0.01, ***P < 0.001.

To investigate the effect of ANXA7 knockdown on COX2 and PGE_2_, cultured HESCs were transfected with non-targeting (NT) or siRNA targeting ANXA7 and then decidualized for 6 days with CM. qRT-PCR analysis demonstrated that silencing of ANXA7 significantly upregulated *COX2* gene expression compared with the non-targeting control (*P* < 0.05; Figure 3A). Western blot results indicated that loss of ANXA7 upregulated COX2 protein level significantly (Figure 3B and Supplementary Figure S3). To test whether loss of ANXA7 increases PGE_2_ levels, HESCs were transfected with NT or siRNA targeting *ANXA7* and then decidualized for 6 days. Cell culture supernatants were collected and PGE_2_ levels measured using an ELISA kit. As shown in Figure 3C, loss ANXA7 increased PGE_2_ levels significantly (*P* < 0.001).

### Loss of AnxA7 Enhances Receptivity

**FIGURE 4:**
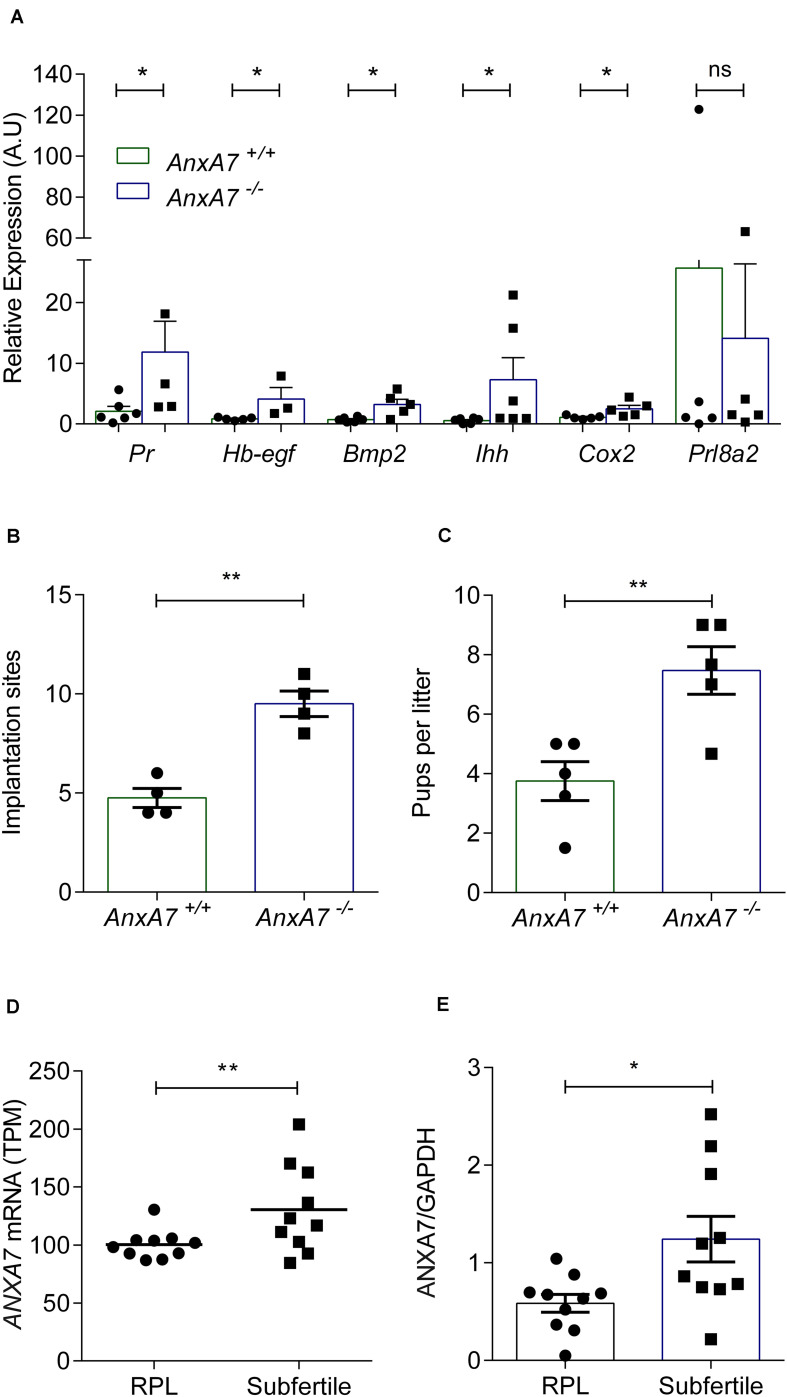
Loss of ANXA7 enhances receptivity. **(A)** Mice were pseudo-mated and the uteri collected at day 5.0 d.p.c. Arithmetic means ± SEM (*n* = *AnxA7*^+/+^; 6 and *AnxA7*^–/^*^–^* 5) of murine transcripts of *Pr* (Progesterone receptor), *Hb-egf* (Heparin binding -Egf), *Bmp2* (Bone morphogenetic protein), *Ihh* (Indian hedgehog), *Cox2* (Cyclooxygenase 2) and *Prl8a2* (Decidual PRL-related protein) in *AnxA7* knockout mice (*AnxA7*^–/^*^–^*) and wild type littermates (*AnxA7*^+/+^). *AnxA7^–/–^* female mice crossed with wild type males and wild type females with *AnxA7^–/–^* males. Arithmetic means ± SEM of **(B)** number of implantation sites at 8.5 d.p.c. (*n* = 4) and **(C)** pups per litter (*n* = 5). **(D)** Human *ANXA7* mRNA levels (GEO 65102) in RPL and Subfertile patients (*n* = 10). **(E)** Arithmetic means ± SEM (*n* = 10) of ANXA7 protein abundance in RPL and Subfertile patients patient samples. Demographic and clinical characteristics are presented in Supplementary Tables S1, S2. Student’s *t*-test was used to calculate statistical significance where *P < 0.05 and **P < 0.01.

In view of its role in PG synthesis, ANXA7 could be a putative regulator of endometrial receptivity. We first mined publicly available microarray data that profiled gene expression in periimplantation mouse uterine luminal epithelium (GEO ID: GSE44451). As seen in Supplementary Figure S4, *AnxA7* levels in wild type (WT) mice significantly increased upon transition of the receptive to the refractory phase (*P* < 0.05). To define the role of AnxA7 in regulating endometrial receptivity, pseudopregnancy was induced in *AnxA7*^–/–^ and WT (*AnxA7*^+/+^) female mice. Uterine horns were collected 5.0 days post coitus (dpc) and total RNA was extracted. The number of oocytes were the same in both groups (data not shown), thus ruling out “hyper-ovulation.” As demonstrated in Figure 4A, several murine endometrial receptivity genes were up-regulated in the *AnxA7****^–^***^/^***^–^*** female mice, including *Cox2* transcripts. Decidualization marker *Prl8a2* tended to be lower in *AnxA7****^–^***^/^***^–^*** female mice, although the difference did not reach statistical significance (*P* = 0.06). We further tested if loss of AnxA7 impacts on the number of implantation events and litter size. As seen in Figure 4, there were significantly more implantation sites (Figure 4B) and increased litter sizes knockout compared to WT mice (Figure 4C).

### ANXA7 Regulates Endometrial Receptivity in Human Endometrium

To explore further the putative link between ANXA7 expression and endometrial receptivity, we explored midluteal endometrial RNA-sequencing data obtained from 10 subfertile patients and 10 women with a history of RPL (GEO Profiles ID: GSE65102). RPL is associated with a prolonged window of implantation, out-of-phase implantation, and heightened receptivity (i.e., superfertility), defined by short time-to-pregnancy (Teklenburg et al., 2010b; Salker et al., 2012a; Lucas et al., 2020; Ticconi et al., 2020). As shown in Figure 4D, endometrial *ANXA7* transcript levels were significantly higher in subfertile patients when compared to RPL subjects (Supplementary Table S1). The difference in endometrial ANXA7 protein expression between the clinical groups was also confirmed by western blot analysis (Figure 4E and Supplementary Figure S5 and Supplementary Table S2).

## Discussion

Annexin A7 is a member of the calcium dependent phospholipids binding proteins and influences many functions and metabolic processes. In this study we reveal that ANXA7 is upregulated in the secretory phase of human endometrium and that *ANXA7* mRNA and protein levels are significantly upregulated in the HESCs decidualized with 8-Br-cAMP and MPA. We also show that endometrial cells express the 51 kDa isoform only. RNA-seq data on midluteal endometrial biopsies showed that *ANXA7* transcript levels varies between 90 and 204 transcripts per million, indicating moderate to high expression (Lucas et al., 2016) when compared to other tissues (Fagerberg et al., 2014). The present observations further show that receptivity is higher and litter size larger in gene-targeted female mice lacking functional AnxA7 (*AnxA7^–/–^*) when compared to WT (*AnxA7^+/+^*) mice. Finally, we show that endometrial *ANXA7* transcript and protein levels are significantly lower in RPL compared to subfertile patients. Importantly, RPL patients are more fertile, i.e., receptive toward the implanting embryo, than healthy women (Ticconi et al., 2020). The present study thus uncovers a novel molecular determinant of endometrial receptivity.

In response to elevated circulating progesterone levels and rising cellular cAMP levels, differentiation of the decidua takes place. Differentiating HESCs must transit through two distinct functional phenotypes for implantation to take place (Salker et al., 2012b; Lucas et al., 2013; Gellersen and Brosens, 2014). This decidual transitional pathway is characterized first by an acute auto-inflammatory phase, which is then followed by a profound anti-inflammatory response. The initial pro-inflammatory response renders the endometrium receptive to embryo implantation (Al-Sabbagh et al., 2011; Salker et al., 2012b). It is interesting to note that the levels of *ANXA7* transcripts in HESCs remain low during the initial pro-inflammatory decidual phase but rise sharply upon the emergence of specialist decidual cells around day 6 of the time-course, suggesting a role in limiting decidual inflammation and closure of the putative implantation window. This conjecture is in keeping with the mouse data. Together, these data point to a role of ANXA7 in participating in closing of the implantation window (Supplementary Figure S6).

Studies from our group and others demonstrated that RPL is associated with an impaired decidual response and mount a prolonged and highly disordered pro-inflammatory response (Salker et al., 2012b; Lucas et al., 2016). Emerging evidence indicates that abnormal decidualization impairs the “embryo-selectivity-checkpoint,” which renders the endometrium excessively permissive to implantation (super-fertile or super-receptive) but unable to sustain the conceptus, thus leading to pregnancy loss (Teklenburg et al., 2010a, b; Sandra et al., 2011; Brosens et al., 2014; Macklon and Brosens, 2014). The current data also supports that the loss of ANXA7 impairs the expression of key decidual genes as well as an increased level of inflammation (PGE_2_). In mice, during implantation, COX2 transiently emerges in stromal cells supporting implantation (Sucurovic et al., 2020). Therefore, it is possible that in the endometrium, in the absence of ANXA7, increased levels of COX2 temporarily create microenvironment suitable for embryo implantation with the synchronized increase of pro-implantation genes. In our murine model, we show that the loss of AnxA7 is associated with an increase of key receptivity genes such as, *Bmp2, Hb-egf, Ihh*, and *Cox2* at 5 dpc. Levels of *Prl8a2* tended to be lower, indicating an impaired decidual response. These data point to a role of AnxA7 in mice model modulating the window of implantation (Wang and Dey, 2006; Salker et al., 2012b).

Prostaglandin E_2_ has a profound effect on embryo development, hatching, and embryo implantation (Niringiyumukiza et al., 2018). ANXA7 is at least partially effective by downregulation of COX2 and inhibition of PGE_2_ formation. In mouse models, deficiencies of cytosolic PLA_2_, COX2, or the use of prostaglandin inhibitors lead to several implantation defects (Pakrasi and Dey, 1982; Psychoyos et al., 1995; Salleh, 2014). Consistent with this hypothesis, re-addition of PGE_2_ or the use of prostaglandin receptor agonists restores or increases implantation rates (Lim et al., 1999; Niringiyumukiza et al., 2018). In our results, we have shown that loss of AnxA7 resulted in a significant increase in the number of implantation sites and numbers of pups born. Furthermore, reduced or impaired endometrial prostaglandin synthesis is associated with dysregulated endometrial receptivity in women with repeated IVF failure (Achache et al., 2010). In keeping with this, we show that at transcript and protein levels ANXA7 was higher in subfertile patients, at least when compared to RPL subjects.

Insulin resistance associated with obesity or polycystic ovary syndrome affects endometrial receptivity, resulting in subfertility (Schulte et al., 2015). *In vivo*, AnxA7 deficiency was shown to decrease the insulin sensitivity of cellular glucose uptake and decreases Sodium/glucose cotransporter 1 (Sglt1) activity in the jejunum lowering glucose absorption and these effects were virtually abrogated by inhibition of COX2 with aspirin (Luo et al., 2015). Thus, the use of aspirin in treating implantation failure in those with insulin resistance is promising. However, clinical trials in the use of aspirin for the use implantation failure remain controversial (Fatemi and Popovic-Todorovic, 2013).

Annexin A7 is required for the stimulation of gastric acid secretion by glucocorticoids and thus contributes to the upregulation of gastric acid secretion by stressful situations (Pasham et al., 2013). It is tempting to speculate that ANXA7 is similarly required for the down-regulation of endometrial prostaglandin synthesis by glucocorticoids (Bazer et al., 2010) and thus, in co-operation with glucocorticoids, closes the receptive window during physical and psychological stressful situations. Glucocorticoid excess following stressful situations is a well-known cause of infertility (Rooney and Domar, 2016), which is, however, at least in part due to derangement of gonadotropin release (Iwasa et al., 2017). Further experiments are required to validate this hypothesis.

Several implantation events, including blastocyst-endometrium adhesion, growth factor and transcription factor signaling are regulated by Ca^2+^ signaling. When intracellular Ca^2+^ levels rise ANXA7 can redistribute itself to the plasma membrane and interact with PLA_2_ (Herr et al., 2003). Further, ANXA7 is involved in the regulation of intracellular Ca^2+^ homeostasis in several cell types (Voelkl et al., 2014). In endometrial cells, impaired intracellular calcium signaling can decrease the expression of Ca^2+^-responsive implantation genes such as COX2, WNT4, and BMP2 resulting in implantation failure (Salker et al., 2018). Moreover, in calcium-dependent-exocytotic secretory processes, ANXA7 -GTPase activity is increased (Herr et al., 2001; Watson et al., 2004). The joint contribution of GTP and Ca^2+^ increases membrane fusion and secretion (Herr et al., 2001; Watson et al., 2004). It is tempting to speculate that ANXA7 may interact with its Ca^2+^-binding partners and is able to fine-tune intracellular Ca^2+^ levels, thus regulating various Ca^2+^-dependent cellular processes essential for implantation. Deregulation of this balance may lead to reproductive failure. Clearly further studies are required to decipher these pathways.

Our results also reveal that loss of ANXA7 impaired decidualization, but promoted an environment conducive for implantation presumably in part by augmenting COX2 levels and thus PGE_2_ formation. Altered endometrial COX2 activity has been implicated in compromised fertility following several disorders (Vilella et al., 2013). It is noteworthy to point out that the 129/SVJ/*AnxA7^+/+^* (WT) mice are known to be poor breeders (Festing and Blackmore, 1971); and that the loss of *AnxA7* increases pup numbers significantly. The present observations, however, do not rule out the contribution of other mechanisms resulting up-regulation of COX2 and enhanced PGE_2_ formation on receptivity in *AnxA7* deficient mice.

In conclusion, ANXA7 is expressed in human endometrium and upregulated during decidualization. Genetic knockout of *AnxA7* leads to increased litter size in mice potentially by extending the window of implantation. Low *ANXA7* transcript levels are observed in women suffering from RPL. Thus, ANXA7 participates in the orchestration of endometrial receptivity and its deranged expression compromises female reproduction.

## Materials and Methods

### Cell Culture

Human endometrial stromal cells from Applied Biological Materials Inc (#T0533, Abm, Canada) (Krikun et al., 2004), were maintained at 37°C in a humidified 5% CO_2_ atmosphere in DMEM/F-12 medium (Gibco, United Kingdom) containing 10% (v/v) dextran coated charcoal striped (Sigma, United States) fetal bovine serum (Gibco, Germany), 1% (v/v) antibiotic-antimycotic solution (Gibco, United States), and 1% (v/v) L-glutamine (Gibco, United Kingdom). Approximately, 200,000 cells were plated in 6-well plates and allowed to grown to (80–85%) confluency for 48 h. Before treatment or transfection, the culture medium was changed to 2% (v/v) dextran coated charcoal striped fetal bovine serum, 1% (v/v) antibiotic-antimycotic solution and 1% (v/v) L-glutamine. The cells were then decidualized with 0.5 μM 8-Bromo-cAMP (8-Bromo-cAMP, Tocris, United Kingdom) and 1 μM Medroxyprogesterone 17-acetate (CM; decidualizing stimulus) (MPA, Sigma, Germany) as indicated. Media was replaced every 48 h with fresh CM-media.

### Transfection Experiments

In confluent cultures, *ANXA7* was silenced by using ON-TARGET plus SMARTpool small interference RNA (siANXA7, L-010760-00-0005; Dharmacon, United States). The siRNA was used at a final concentration of 2.75 nM with VIROMER GREEN Transfection Reagent (Lipocalyx GmbH, Germany) following the manufacturer’s instructions. After incubating the cells with the transfection complex for 6 h, the medium was then discarded and replaced with treatment medium (CM; as described above) and the cells were then cultured for another 6 days.

### Real-Time Quantitative PCR (qRT-PCR)

Total RNA was isolated from cells or (80 mg) tissues using a TRIzol reagent (Thermo Fischer) and transferred to a microcentrifuge tube. After 5 min incubation at room temperature 100 μl chloroform (Roth, Germany) was added and vortexed. The mixture was subsequently centrifuged at 13,000 rpm for 30 min at 4°C. The upper clear part was transferred to a new (RNASE free) microcentrifuge tube. 200 μl 2-propanol (Sigma) was added and mixed by vortexing. After 10 min incubation at room temperature, the sample was centrifuged at 13,000 rpm for 15 min at 4°C. The supernatant was discarded and the pellet air dried for 5 min. To this 50 μl of DEPC treated water (Sigma) was added to dissolve the pellet. The RNA concentration was measured by the nanoplate method (Thermo Scientific). The complementary DNA was synthesized by using Maxima H Minus cDNA synthesis kit (Thermo Scientific). qRT-PCR was then performed with PowerUp SYBR Green Master Mix (Thermo Scientific) using gene-specific primers purchased from Sigma. Primers were designed using primer blast (NCBI; Supplementary Method 1). For housekeeping controls; *L19* was used for human sample and *Cyclo* for mice samples. The expression levels of the samples are provided as arbitrary units defined by the ^ΔΔ^Ct method. All measurements were performed in duplicate. Melting curve analysis and agarose gel electrophoresis confirmed amplification specificity.

Human primers used for qRT-PCR:

**Table:** 

Gene	Primer sequence (5′–3′)
***L19***	F: GCGGAAGGGTACAGCCAAT R: GCAGCCGGCGAAA
***AnxA7***	F: CTGCTGGGTCAGAATGTCATA R: AGGAGGATATCCAGGGAAAGGT
***COX2***	F: GCTCAAACATGATGATGTTTGCATTC R: GCTGGCCCTCGCTTATGA
	

Murine primers used for qRT-PCR:

**Table:** 

Gene	Primer sequence (5′–3′)
***Cyclo***	F: TGGAGAGCACCAAGACAGACA R: TGCCGGAGTCGACAATGAT
***Pr***	F: GGTGGGCCTTCCTAACGAG R: GACCACATCAGGCTCAATGCT
***Ihh***	F: GCTTCGACTGG\]GTGTATTACG R: GCTCGCGGTCCAGGAAAAT
***Hb-egf***	F: CTTGCGGCTACTTGAACACA R: GAAAGCAGGATCGAGTGAGC
***Bmp2***	F: GGGACCCGCTGTCTTCTAGT R: TCAACTCAAATTCGCTGAGGAC
***Prl8a2***	F: TGCTCAGATCCCCTTGTGAT R: AGCTGGTGGGTTTGTGACAT

### Western Blotting

Total protein samples were prepared by lysing the adherently cultured cells in a lysis buffer containing 0.5 M Tris hydrochloride (Roth) pH 6.8, 20% Sodium dodecyl sulfate (SDS, Sigma), 0.1% Bromophenol blue (Serva),1% beta mercaptoethanol (Sigma), and 20% glycerol (Roth). Tissue samples were extracted using the previous method (Salker et al., 2011). Equal amount of total protein was separated by sodium dodecyl sulphate-polyacrylamide gel electrophoresis (SDS-PAGE) and transferred onto polyvinylidene fluoride membranes (PVDF, GE Healthcare Life Sciences, Germany) as previously described (Salker et al., 2011). After blocking in 5% non-fat milk (Roth, Germany) in a Tris buffered saline (TBS-T) containing 0.1% Tween-20 (Sigma) for 1 h at room temperature. The membranes were then incubated overnight with primary antibodies AnxA7 (#3666; Cell Signaling, Netherlands), COX2 (#aa570-598; Cayman Chemical Company), GAPDH (#2218L; Cell Signaling) in the blocking buffer at 4°C overnight. The following day the primary antibody was removed and the membrane was washed four times with TBS-T each for 10 min. The membranes were then incubated with appropriate secondary antibodies in the blocking buffer at room temperature for 1 h followed by four washes with TBS-T. Chemiluminescent detection kit (WesternBright^TM^ ECL, Advansta, United States) used for the visualization of the protein complexes. The fluorescence signals were scanned with an iBright CL1000 (Thermo Scientific), and the intensities were assessed by a densitometry analysis to measure the relative expression of the target proteins using GAPDH as a control by ImageJ software. All samples are normalized with the control (Taylor and Posch, 2014).

### Immunofluorescence

Human endometrial stromal cells (1000 cells) were plated on glass chamber slide and grown for 48 h. Treatment with CM was performed as described above for transfected and non transfected samples. Post treatment the cells were fixed for 15 min with 4% paraformaldehyde, washed with PBS, and permeabilized for 10 min in 0.1% Triton X-100/PBS. The slides were blocked with 5% BSA in 0.1% TritonX-100/PBS for 1 h at room temperature. Cells were stained for actin with eflour660-phalloidin (1:1000, #50655905) for 1 h at room temperature. The slides were mounted with ProLong Gold antifade reagent with DAPI (#P36931, Invitrogen). Microscopy was performed with an EVOS M7000 cell imaging system (Thermo Fischer) with an Apochromat 0.4 NA cover slip corrected × 10 objective. Scale bar was 100 μm.

### ELISA

After treatment of the HESCs as stated above culture media were harvested and stored at *−*80°C. ELISA performed by using Human Prostaglandin E_2_ ELISA Kit (Invitrogen) and Human Prolactin/PRL ELISA Kit (Abcam) following the manufacturer’s instructions.

### Animal Experiments

Experiments were performed in gene-targeted 129/SVJ mice lacking *AnxA7* (*AnxA7^–/–^*) and in corresponding wild type mice (*AnxA7^+/+^*). Generation, properties and genotyping of *AnxA7^–/–^* mice were described earlier (Herr et al., 2003). All animal experiments were conducted according to the German law for the care and use of laboratory animals and were approved by the local ethics committee. The mice (age 8–12 weeks) were fed a normal diet and had access to drinking water *ad libitum*. The mice were kept under constant humidity (55 ± 10%), temperature (22 ± 2°C) and 12 h light-dark cycle conditions. The, *n*, number was calculated using a power calculation. Implantation sites were counted as previously described (Salker et al., 2012a).

### Patient Selection and Sample Collection

The study was approved by the National Health Service National Research Ethics–Hammersmith and Queen Charlotte’s & Chelsea Research Ethics Committee (1997/5065). Subjects were recruited from the Implantation Clinic, a dedicated research clinic at University Hospitals Coventry and Warwickshire National Health Service Trust. Written informed consent was obtained from all participants in accordance with the guidelines in the Declaration of Helsinki 2000. Samples were collected using a Wallach Endocell sampler (Wallach) under ultrasound guidance as previously described (Salker et al., 2017). Endometrial biopsies were timed between 6 and 10 days after the preovulatory Luteinizing Hormone (LH) surge. Biopsies were collected in ovulatory cycles. None of the subjects were using hormonal treatments for at least 3 months prior to sample collection. The sub-fertility group consisted of women with a history of conception delay due to endometriosis, male factor, tubal factor, PCOS or unexplained infertility. RPL was defined as three or more consecutive pregnancy losses before 24 weeks gestation. Demographic and clinical characteristics are presented in Supplementary Tables S1, S2.

We confirm that all methods performed, including obtaining of consent, were performed in accordance with the relevant guidelines and regulations as approved by the ethics committee.

### Statistical Analysis

Values are presented as means ± SEM. Data were analyzed using the Students *t*-test or 1 way-ANOVA for significance using the Graphpad Prism software (GraphPad software Inc., San Diego, CA, United States). Number of replicates (n). Power calculation for sample size was performed using the G^∗^Power program. Values of *P* ≤ 0.05 were considered significant. Figures presented were made using Graphpad Prism.

## Data Availability Statement

The raw data supporting the conclusions of this article will be made available by the authors upon reasonable request, to any qualified researcher.

## Ethics Statement

The studies involving human participants were reviewed and approved by the National Health Service National Research Ethics–Hammersmith and Queen Charlotte’s & Chelsea Research Ethics Committee (1997/5065). The patients/participants provided their written informed consent to participate in this study. The animal study was reviewed and approved by the Regierungspräsidium Tübingen, Baden-Württemberg, Germany (35/9185.82-2 F01/19M).

## Author Contributions

MS and FL: conceptualization. MA, MS, AU, AW, JR, TO, and YS: methodology, formal analysis, and data curation. SB, DW, JB, and FL: resources or patient recruitment. MS: supervision and project administration. MS, SB, DW, and JB: funding acquisition. MA, MS, AU, JR, TO, YS, AW, SB, DW, JB, and FL: writing – original draft and writing – review and editing. All authors reviewed the manuscript and have approved its submission and publication.

## Conflict of Interest

The authors declare that the research was conducted in the absence of any commercial or financial relationships that could be construed as a potential conflict of interest.
