# Supplementary material for: Annexin A7 Regulates Endometrial Receptivity
Source: Front Cell Dev Biol. 2020 Aug 14;8:770. doi: 10.3389/fcell.2020.00770 (PMC7456953; doi:10.3389/fcell.2020.00770)
Supplement: Supplementary file 1 [file Data_Sheet_1.PDF]

Supplementary Information for

Annexin A7 regulates endometrial receptivity

Md Alauddin[1], Madhuri S Salker[1*], Anja Umbach[2], Janet Rajaxavier[1], Toshiyuki Okumura[1,3], Yogesh Singh[4], Anna Wagner[1], Sara Y Brucker[1], Diethelm Wallwiener[1], Jan J Brosens[5,6] and Florian Lang[2*]

[1]Department of Women's Health, Eberhard-Karls University Tuebingen, Calwerstr 7, 72076 Tuebingen, Germany.
[2]Department of Physiology, Eberhard-Karls University Tuebingen, Wilhelmstr 56, D-72076 Tuebingen, Germany.
[3]Department of Obstetrics and Gynecology, Juntendo University School of Medicine, Tokyo, Japan
[4]Institute for Medical Genetics and Applied Genomics, Eberhard-Karls University Tuebingen, Calwerstr 7, 72076 Tuebingen, Germany.
[5]Division of Biomedical Sciences, Warwick Medical School, CV2 2DX Coventry, United Kingdom.
[6]Tommy's National Centre for Miscarriage Research, University Hospitals Coventry and Warwickshire NHS Trust, Clifford Bridge Rd, Coventry CV2 2DX, United Kingdom

This PDF file includes:

Tables S1 & S2
Figures S1 –S6
Supplementary Method 1

Table S1: Patient demographics and characteristics (Figure 4d -RNA-seq).

	Age	Live Birth	Number of Losses	BMI	LH+ Day
**Sub fertility**	36.6 ± 1.36	0	0	24.6 ± 1.42	7.2 ± 0.13
**RPL**	37.5 ± 0.79	0.3± 0.15	4.9 ± 0.52	25.44 ± 1.02	7.9 ± 0.45

Data shown are arithmetic means ± SEM (n = 10 in each group)
Sub fertility; unexplained = 5; male factor = 2; tubal disease = 1; endometriosis = 1; polycystic ovary syndrome = 1.
RPL; Recurrent Pregnancy Loss
BMI; Body Mass Index
LH+; days after the luteinizing hormone peak

Table S2: Patient demographics and characteristics (Figure 4e -Western blot).

	Age	Live Birth	Number of Losses	BMI	LH+ Day
**Sub fertility**	35.2 ± 2.68	0	0	22.4 ± 2.07	9 ± 0.70
**RPL**	36.4± 3.28	0	4± 1.22	24.5± 3.10	8.2 ± 1.30

Data shown are arithmetic means ± SEM (n = 10 in each group)
Sub fertility; Unexplained = 10
RPL; Recurrent Pregnancy Loss = 10
BMI; Body Mass Index
LH+; days after the luteinizing hormone peak

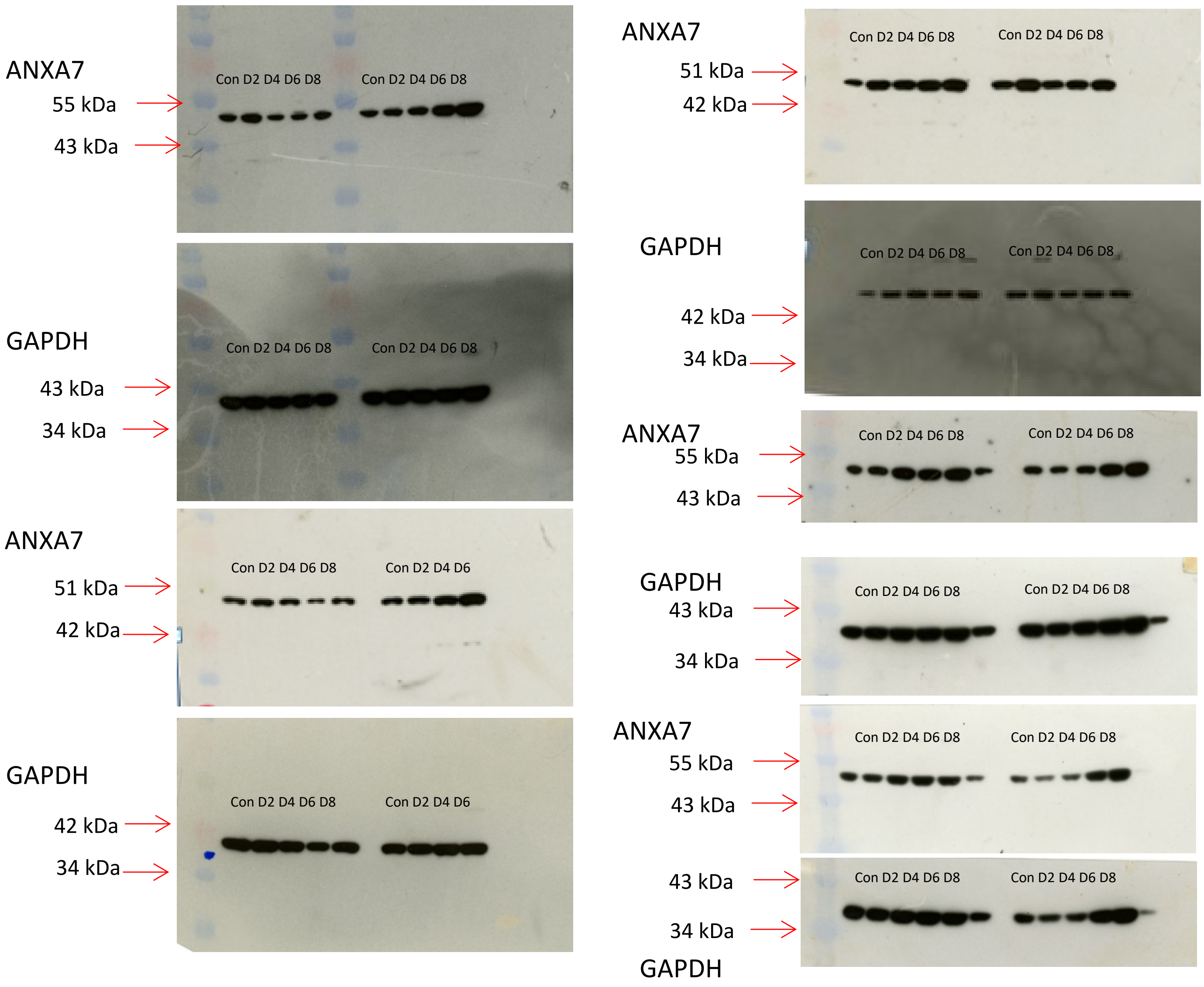

Figure Sup 1: ANXA7 level in Human Endometrial Stromal Cells (HESCs).
Original Western blot of ANXA7 (51 kDa approx.) and GAPDH (37 kDa) as shown in Figure 1c (n = 10).

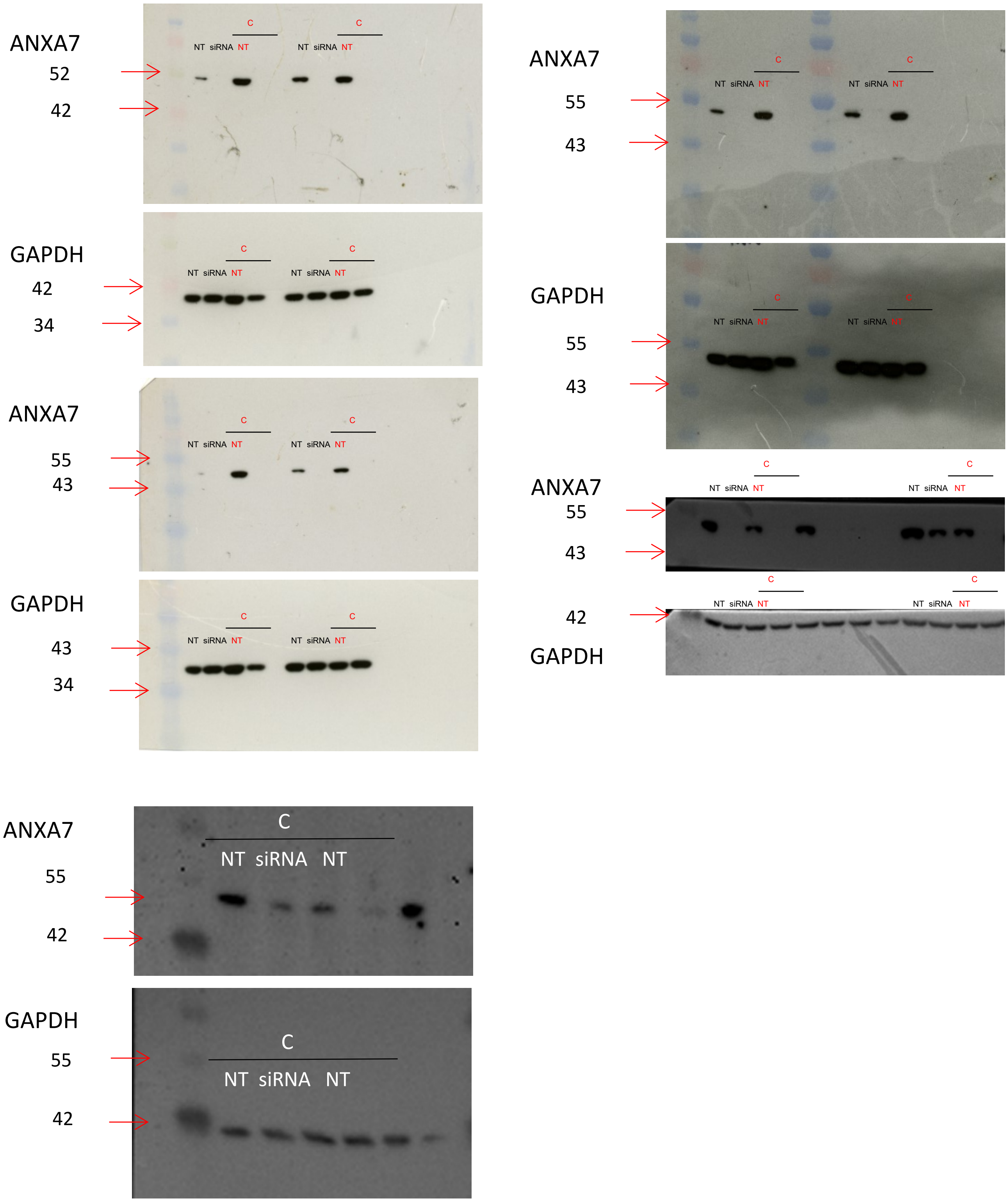

Figure Sup 2: ANXA7 Knockdown in HESCs.

Original Western blot of ANXA7 and GAPDH as shown in Figure 2b (n = 10).

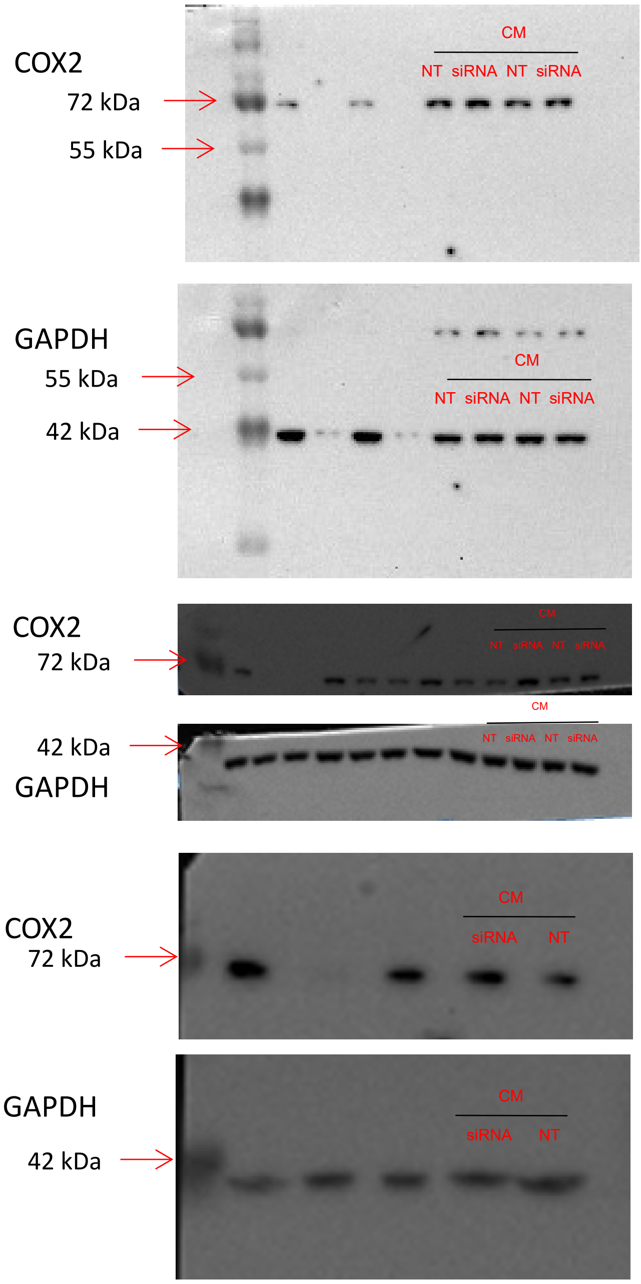

Figure Sup 3: COX2 level in ANXA7 Knockdown HESCs.

Original Western blot of Cox2 and GAPDH as shown in Figure 3b (n = 5).

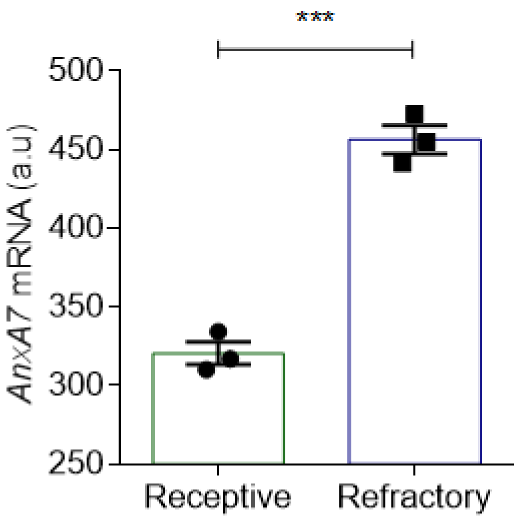

Figure Sup 4: Endometrial AnxA7 transcripts during the mouse implantation window. Comparison of endometrial *AnxA7* transcripts during the mouse implantation window Transcript levels are expressed as arbitrary units (a.u). The data were derived from *in silico* analysis of publicly available microarray data [Gene Expression Omnibus (GEO) Profiles; ID: GSE44451]. (n = 3), ***P<0.0001 using Student's t-test.

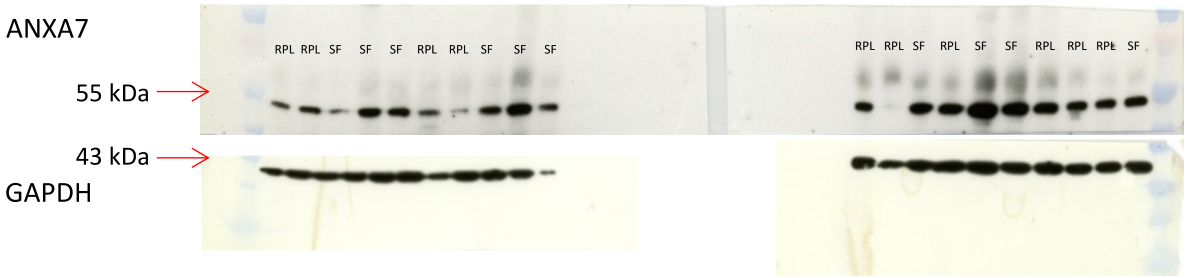

Figure Sup 5: Infertility is associated with reduced endometrial ANXA7

Original Western blot of ANXA7 expression in mid-luteal endometrial biopsies from Subfertile (SF; n = 10) and recurrent pregnancy loss patients (RPL; n = 10).

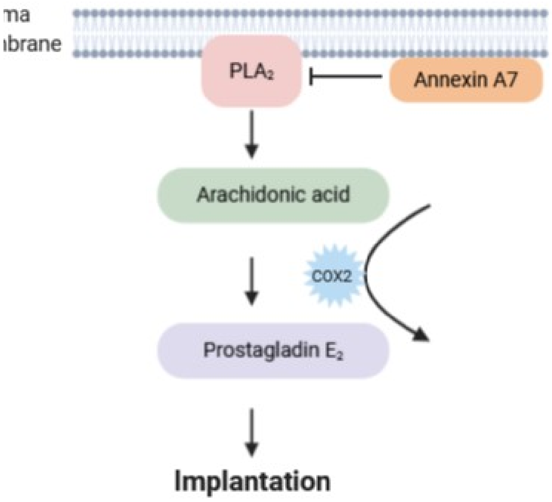

Figure S6: ANXA7 regulates embryo implantation
PLA_2 generates arachidonic acid (AA) from phospholipids. AA can then generate prostaglandin E_2 by the enzymatic activity of COX2. Here we show that the Annexin A7 inhibits the PLA_2 activity and subsequently prevents implantation.

Supplementary Method 1

Homo sapiens *ANNEXIN A7* (*ANXA7*), transcript variant 2, mRNA
NCBI Reference Sequence: NM_004034.4
ORIGIN

1 atcttgcggg agaccgggtt gggctgtgac gctgctgctg gggtcagaat gtcataccca
61 ggctatcccc caacaggcta cccacctttc cctggatatc ctcctgcagg tcaggagtca
121 tcttttcccc cttctggtca gtatccttat cctagtggct ttcctccaat gggaggaggt
181 gcctacccac aagtgccaag tagtggctac ccaggagctg gaggctaccc tgcgcctgga
241 ggttatccag cccctggagg ctatcctggt gccccacagc caggggggagc tccatcctat

Product length 71bp

F: 5'CTG CTG GGG TCA GAA TGT CAT A 3'
R: 3'AGG AGG ATA TCC AGG GAA AGG T 5'
